# Low fT3 Syndrome, Dialysis Inadequacy, and Death Occurrence in Hemodialysis Patients: Evidence of a Vicious Circle from a Prospective Bi-Center Observational Study

**DOI:** 10.3390/jcm15062400

**Published:** 2026-03-21

**Authors:** Aleksandra Młodożeniec, Małgorzata Rodzoń-Norwicz, Renata Orłowska-Florek, Krystyna Tęcza, Piotr Młodożeniec, Krzysztof Gargasz, Agnieszka Gala-Błądzińska

**Affiliations:** 1 Clinic of Internal Medicine, Nephrology and Endocrinology with Nuclear Medicine Laboratory and Dialysis Center, State Hospital 2, Lwowska Street 60, 35-301 Rzeszów, Polandagala.edu@gmail.com (A.G.-B.); 2 Department of Human Physiology, Faculty of Medicine, University of Rzeszów, al. Tadeusza Rejtana 16C, 35-959 Rzeszów, Poland; 3 Department of Nephrology and Endocrinology, Faculty of Medicine, University of Rzeszów, al. Tadeusza Rejtana 16C, 35-959 Rzeszów, Poland; 4 Department of Nephrology and Dialysis, Dystrict Hospital John Paul II, Grunwaldzka 4, 36-100 Kolbuszowa, Poland; 5 ProVital Medical Center, Plac Wolności 17, 35-001 Rzeszów, Poland; 6 Faculty of Medicine, Data Processing Laboratory, Natural and Medical Center for Innovative Research, University of Rzeszów, al. Tadeusza Rejtana 16C, 35-959 Rzeszów, Poland; 7 Clinic of Nephrology and Dialysis Unit, Fryderyk Chopin University Clinical Hospital, ul. Fryderyka Szopena 2, 35-055 Rzeszów, Poland

**Keywords:** non-thyroidal illness syndrome, hemodialysis, Kt/V, thyroid hormones, number of deaths

## Abstract

**Background/Objectives:** Non-thyroidal illness syndrome (NTIS) also known as low FT3 syndrome is characterized by altered thyroid hormone levels during severe illness, is common in end-stage renal disease, and reflects metabolic and inflammatory stress. This study evaluated the thyroid hormone profiles of patients undergoing maintenance hemodialysis, assessing relationships between NTIS severity and dialysis adequacy while accounting for mineral and bone metabolism markers, anemia status, duration of dialysis therapy, and their association with the number of deaths during follow-up. **Methods**: This prospective bi-center study included adults receiving maintenance hemodialysis for at least 3 months. Patients treated for thyroid disease or taking medications affecting the hypothalamus–pituitary–thyroid axis were excluded. Thyroid-stimulating hormone, free triiodothyronine (fT3), and free thyroxine (fT4) levels were measured, and dialysis adequacy was assessed using spKt/V. Patients were classified as euthyroid or having NTIS (stratified by severity), and associations between clinical characteristics and the number of deceased patients during a 6-month observation period were analyzed using receiver operating characteristic (ROC) curves to determine prognostic cut-off values for thyroid hormones. **Results**: Among 74 patients, 50% had NTIS and exhibited significantly lower dialysis adequacy than euthyroid individuals (median spKt/V 1.0 vs. 1.1; *p* = 0.03), with spKt/V declining as NTIS severity increased (stages I–III, *p* = 0.008). NTIS severity correlated with age and pulmonary comorbidities, while mineral and bone metabolism markers were comparable between the groups. During the 6-month follow-up, 23% of the patients died, exhibiting significantly lower fT3 and fT4 levels than survivors. ROC analysis identified clinically relevant fT3 and fT4 cut-off values that were associated with the number of deaths. **Conclusions**: NTIS in hemodialysis patients correlates with reduced dialysis adequacy and appears to be a prognostic factor for risk of death. NTIS severity correlated with declining spKt/V, potentially reflecting disease burden, and thyroid hormone assessment may provide prognostic information.

## 1. Introduction

Non-thyroidal illness syndrome (NTIS), also referred to as “low fT3 syndrome” or “sick euthyroid syndrome”, is characterized by altered thyroid hormone metabolism in the absence of primary thyroid gland disease. This condition manifests in critically ill patients, including those with end-stage renal disease (ESRD) [1,2,3]. Typical temporary hormonal alterations in this pathology include decreased total triiodothyronine (T3) and free triiodothyronine (fT3) levels, alongside low or normal free thyroxine (fT4) and normal or reduced thyroid-stimulating hormone (TSH) levels [2,4]. NTIS pathogenesis is still incompletely elucidated.

Thyroid function is regulated by the hypothalamic–pituitary–thyroid (HPT) axis, which modulates the secretion of thyrotropin-releasing hormone (TRH) and TSH [5]. The mechanisms implicated in alterations within the HPT axis and target tissues in NTIS include TRH suppression in the paraventricular nucleus of the hypothalamus and reduced TSH messenger RNA expression of the β-subunit in the pituitary gland [6]. Pro-inflammatory cytokines are principal factors in NTIS as they can inhibit individual stages of thyroid hormone (TH) production.

In addition to changes in the HPT axis, NTIS is associated with alterations in various target tissues, such as a reduction in the number of TH transporters, decreased expression of thyroid hormone receptors, and diminished deiodinases activity [1,6,7].

The interrelationship between the thyroid and kidneys is well documented. Thyroid disorders induce changes in glomerular and tubular functions, as well as in electrolyte and water homeostasis [8]. TH impacts the local renin–angiotensin system, stimulates the release of renin by juxtaglomerular cells, and influences kidney angiotensinase activity. Alterations in angiotensin metabolism may contribute to changes in renal function and are associated with thyroid disorders [9].

Del Compare et al. proposed that renal dopamine (DA) synthesis is partially dependent on thyroid hormone levels and that DA receptor responsiveness is altered by TH deficiency [10]. TH acts on the sodium–potassium ATP pump and influences potassium permeability in the proximal tubule membranes of the kidney [11,12].

The kidneys play a crucial role in iodine and TH metabolism [8,13]. In humans, approximately 80% of T3 is produced through peripheral conversion by type 1 (liver and kidney) or type 2 (skeletal muscle) deiodinases, and considerable amounts of T4 and T3 are activated in kidneys [14]. Chronic kidney disease (CKD) leads to substantial variations in TH levels, commonly resulting in subclinical primary hypothyroidism and NTIS [15,16,17,18,19,20,21], and excess serum iodine favors hypothyroidism development in those patients [8,22].

NTIS occurrence increases with CKD progression, reaching up to 80% in ESRD [3], and ESRD may result in significant changes in TH metabolism. For instance, loss of TH-binding proteins during dialysis, reduced muscle mass (and expression of deiodinase type 2), direct inhibitory effects of pro-inflammatory cytokines on the thyroid gland, chronic metabolic acidosis, malnutrition, and selenium deficiency were observed in patients with ESRD [23,24,25,26,27,28,29,30].

Elevated reverse T3 levels have been documented in classic NTIS [2,4]. In patients with uremia, NTIS can manifest without an elevation in reverse T3 (rT3) levels [8]. Kaptein et al. attributed this phenomenon to the redistribution of rT3 from the vascular space to the extravascular space and increased cellular uptake of rT3 [24,31].

The prevailing understanding and consensus among researchers is that NTIS represents an adaptive, protective response of the body aimed at reducing metabolism and conserving energy during illness [1,2,4]. However, in patients with ESRD, NTIS may manifest as a decline in metabolic status and physical performance [32,33,34].

Research indicates that TH concentrations, particularly FT3 levels, may serve as independent predictors of mortality in dialysis patients [23,35,36].

Sciacchitano et al. proposed a method that could aid in assessing the efficacy of T3 treatment in NTIS patients [4], and there have also been efforts to administer levothyroxine, N-acetylcysteine, and sodium bicarbonate to patients with NTIS and CKD [37,38]. However, data regarding the potential benefits of any treatments in NTIS patients are limited.

Therefore, in this study, we aimed to evaluate the thyroid hormone profiles of patients undergoing hemodialysis and to assess the association between NTIS, including its severity and dialysis adequacy, anemia status; response to erythropoietin preparations; dialysis duration; and disturbances in mineral and bone metabolism markers. Additionally, we examined the relationship between TH levels and their association with the number of deceased patients during observation, seeking to identify clinically relevant cut-off values of serum fT3 and fT4 levels associated with risk of deaths in the hemodialysis population.

## 2. Materials and Methods

### 2.1. Study Design

This prospective observational study was conducted at two dialysis centers located in the Subcarpathian region of Europe. It adhered to the principles outlined in the Declaration of Helsinki, and written informed consent was obtained from all participants prior to their enrollment.

### 2.2. Study Population

Adult patients (≥18 years) undergoing a maintenance hemodialysis regimen for at least 3 months were screened for inclusion. Only patients who provided informed consent were included. 

Patients were excluded if they had a known history of thyroid disease (including overt or subclinical hypothyroidism or hyperthyroidism), pituitary disorders, or previous thyroidectomy or were receiving medications known to influence the HPT axis, such as glucocorticoids or TH replacement therapy. Additional exclusion criteria were a dialysis vintage of less than 3 months and severe clinical conditions, including an active inflammatory state, active malignancy or severe liver disease.

Among the analyzed patient groups, cardiac diseases included coronary artery disease, conditions after myocardial infarction or coronary angiography, heart failure, arrhythmias, and conditions after cardiac surgery. Patients with concomitant lung diseases included those with chronic obstructive pulmonary disease or bronchial asthma.

**Figure 1:**
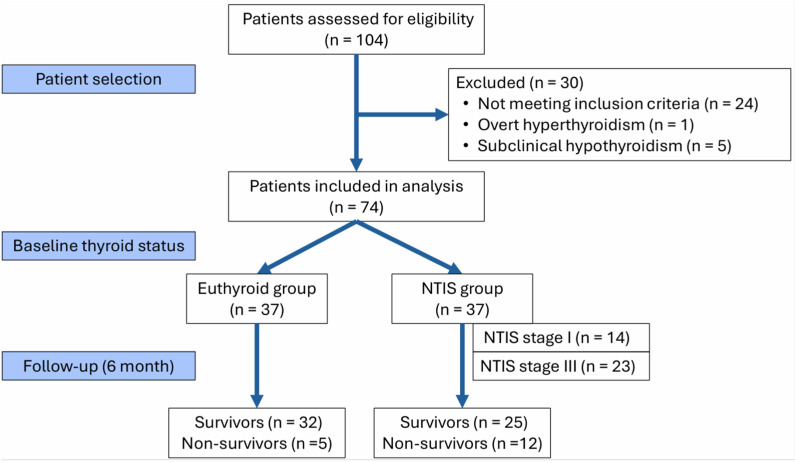
Study flowchart.

After applying the inclusion and exclusion criteria, data from 74 patients (71% of the initially screened cohort) aged 18–89 years were included in the final analysis (Figure 1).

#### 2.2.1. Clinical and Laboratory Assessments

Demographic data and detailed medical histories were collected at the beginning of the study. All patients underwent a comprehensive clinical examination and laboratory evaluation. Hemodialysis sessions were conducted three times per week, with a duration of approximately 3–4 h per session. Vascular access was established via an arteriovenous fistula in all patients.

Blood samples were collected once at the beginning of the dialysis procedure to determine the serum concentrations of fT3, fT4, TSH, calcium, phosphorus, parathyroid hormone (PTH), hemoglobin (Hb) and anti-thyroid antibodies (anti-TPO and anti-TG), and serum urea and creatinine levels were measured before and after dialysis (Siemens Atellica SCI Analyzer, Malvern, PA, USA; Sysmex Analyzer, Kobe, Japan).

All laboratory analyses were conducted at certified hospitals, where serum fT3, fT4, and TSH concentrations were measured using chemiluminescent immunoassays. The laboratory reference ranges were as follows: fT3: 2.3–4.2 pg/mL, fT4: 0.89–1.76 ng/dL, and TSH: 0.55–4.78 μIU/mL. The detailed biochemical parameters, analytical methods, and reference ranges are listed in Table 1.

During the 6-month follow-up period the number of deceased patients (all-cause mortality) were summarized.

#### 2.2.2. Assessment of Dialysis Adequacy

Dialysis adequacy was evaluated using the single-pool Kt/V (spKt/V) method, which was calculated according to the second-generation Daugirdas formula.

Kt/V = −ln(R − 0.008 × t) + (4 − 3.5 × R) × UF/W,

where *R* represents the post-dialysis-to-pre-dialysis serum urea ratio, *t* denotes the dialysis session duration (hours), *UF* is the ultrafiltration volume (L), and *W* is the post-dialysis body weight (kg). The urea distribution volume (V) was estimated using Watson’s formula.

#### 2.2.3. Classification of Thyroid Status

Patients were categorized into two main groups based on their thyroid hormone profiles:

1. Euthyroid group: Patients with serum TSH, fT3, and fT4 concentrations within the reference ranges;
2. NTIS group: Patients with reduced fT3 levels, further subclassified according to NTIS severity:
  - Stage I NTIS: Reduced fT3 (fT3 < 2.3 pg/mL);
  - Stage II NTIS: Reduced fT3 levels accompanied by reduced TSH levels (TSH < 0.55 μIU/mL);
  - Stage III NTIS: Reduced fT3 and fT4 (fT4 < 0.89 ng/dL) with either normal or reduced TSH levels.

### 2.3. Statistical Analysis

Continuous variables were assessed for normality using the Shapiro–Wilk test, supplemented by visual inspection of histograms and distribution characteristics, and were reported as medians along with their minimum and maximum values. Categorical variables are presented as numbers and percentages. For continuous variables, comparisons between the two independent groups were conducted using the Mann–Whitney U test, while Pearson’s chi-square test was performed for categorical variables. When the expected cell counts were low, a chi-square test with Yates’s continuity correction was applied. Statistical significance was set at *p* < 0.05, and all statistical tests were two-tailed. Additionally, missing data were addressed using pairwise deletion.

Patients were initially classified into the euthyroid or NTIS group based on thyroid function. Subsequent analyses focused on comparisons between the euthyroid and NTIS groups, as well as between NTIS stages I and III. Receiver operating characteristic (ROC) curve analysis was performed to evaluate the prognostic value of serum TSH, fT3, and fT4 levels to discriminate between surviving and deceased patients during follow-up.

Optimal cut-off values were established based on sensitivity and specificity, and the area under the curve (AUC) with corresponding *p*-values was calculated. The ROC analyses were performed to determine potential cut-off values associated with the number of deceased patients during follow-up [Appendix A.1]. Due to differences in data availability across clinical and laboratory variables, the number of patients included in the individual analyses varied and was reported for each comparison. Percentages for categorical variables were calculated using the number of patients in each group as the denominator unless otherwise specified.

This study included all eligible patients undergoing hemodialysis in the center during the study period (*n* = 74). As this was an exploratory observational study, the sample size was determined by the available patient population rather than by a priori power calculations. Thus, the limited sample size and relatively small number of outcome events precluded the performance of multivariate regression analyses. Consequently, all results should be interpreted as univariate associations, and statistical analysis was performed using STATISTICA 13 software (TIBCO Software Inc., Tulsa, OK, USA).

### 2.4. Ethics Approval

This study was approved by the Bioethics Committee of the Regional Medical Chamber (Approval No. 29/2024/B), and the procedures were performed in adherence to the ethical standards of the responsible committee and the Declaration of Helsinki of 1975, as revised in 2000. All participants provided written informed consent, and although personal identification numbers were used to match the datasets, they were anonymized.

## 3. Results

A total of 104 patients on dialysis were screened, of whom 74 met the inclusion criteria and were included in the final analysis. They were then classified into the euthyroid (*n* = 37) or NTIS (*n* = 37) group based on thyroid function, and their baseline demographic and clinical characteristics are presented in Table 2.

No statistically significant differences in age, sex distribution, body weight, body mass index (BMI), prevalence of cardiovascular disease, diabetes mellitus type 2, or pulmonary comorbidities were identified between the groups. However, disturbances in mineral and bone metabolism were prevalent among the study population. Hypocalcemia was observed in 70.3% of patients, while normal calcium concentrations were found in 27.0%; hypercalcemia was rare, occurring in only 1.4% of cases. Serum phosphorus concentrations were within the reference range in 39.2% of the patients and were elevated in 59.5%, whereas no cases of hypophosphatemia were identified. Parathyroid hormone concentrations were elevated in 93.2% of patients, whereas normal levels were observed in 4.1% of patients. Missing data accounted for 1.35% and 2.7% of the calcium and phosphorus measurements and PTH, respectively. The laboratory parameters related to mineral and bone metabolism markers were comparable between the euthyroid and NTIS groups. There were no statistically significant differences in hemoglobin levels between the NTIS group vs. the euthyroid group (*p* = 0.2). Sixty-three of the 74 patients (85%) took erythropoiesis-stimulating agents (ESAs), and higher doses of the preparation were observed among patients with NTIS. Longer durations of dialysis were observed in the NTIS group (*p* = 0.3) compared with the euthyreosis group. However, these correlations were not statistically significant, which could be attributed to the small cohort size.

**Figure 2:**
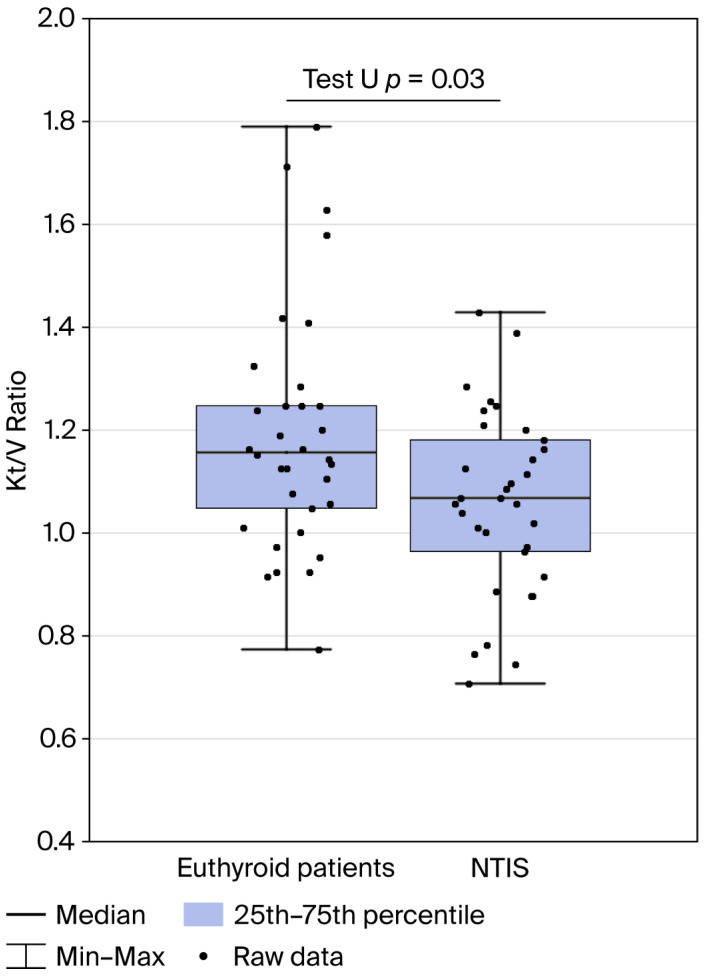
spKt/V ratio in patients with ESRD: euthyroid patients compared with NTIS patients. Mann–Whitney U test, *p* = 0.03.

The spKt/V ratio was significantly lower in patients with NTIS than in euthyroid patients (*p* = 0.03), and this difference is illustrated in Figure 2.

The NTIS cohort was further stratified into stage I (*n* = 14), stage II (*n* = 0) and stage III (*n* = 23) groups, based on disease severity. Comparisons between these subgroups are presented in Table 3. Patients with stage III NTIS were significantly younger than those with stage I NTIS (*p* = 0.01). Pulmonary comorbidities were less frequent in NTIS stage III (*p* = 0.01). Among patients with NTIS III the median time dialysis duration was longer than in the NTIS I group (48 vs. 29.5 months), (*p* = 0.7). There was no statistically significant differences in the hemoglobin levels between the two groups (*p* = 0.5).

Significant differences were observed in urea concentrations before and after hemodialysis as well as in the spKt/V ratio (Table 3).

During the 6-month follow-up period, 17 patients (23%) died, and the comparisons between survivors and non-survivors are presented in Table 4.

**Figure 3:**
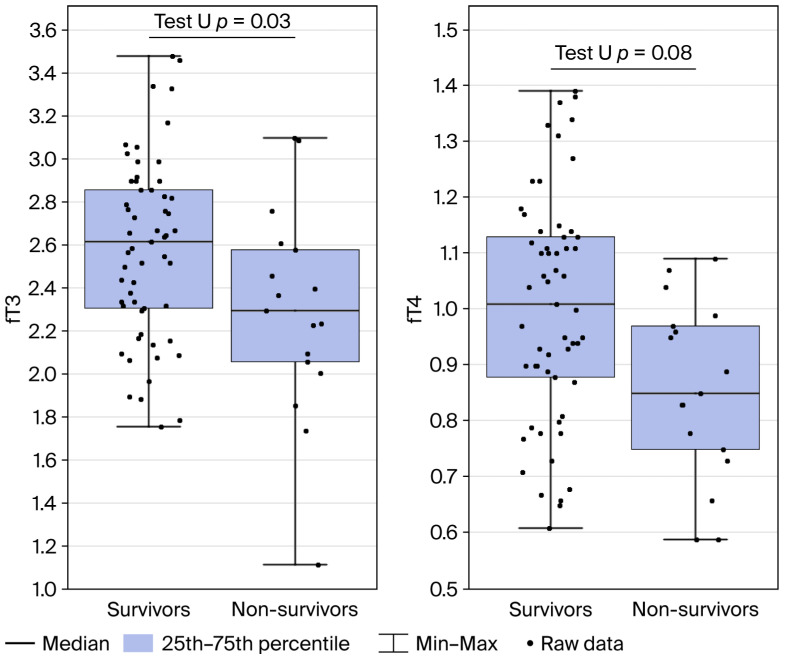
Serum fT3 and fT4 concentrations in survivors and non-survivors. Box plots represent the median, interquartile range (25th–75th percentile), and minimum–maximum values, with individual data points shown. Significant differences between groups were observed for both fT3 (*p* = 0.03) and fT4 (*p* = 0.008) using the Mann–Whitney U test.

Non-survivors exhibited significantly lower serum fT3 (*p* = 0.03) and fT4 (*p* = 0.008) concentrations than survivors, as illustrated in Figure 3. Additionally, dialysis duration was longer among non-survivors compared to survivors group; however, the differences were not statistically significant (*p* = 0.2).

**Figure 4:**
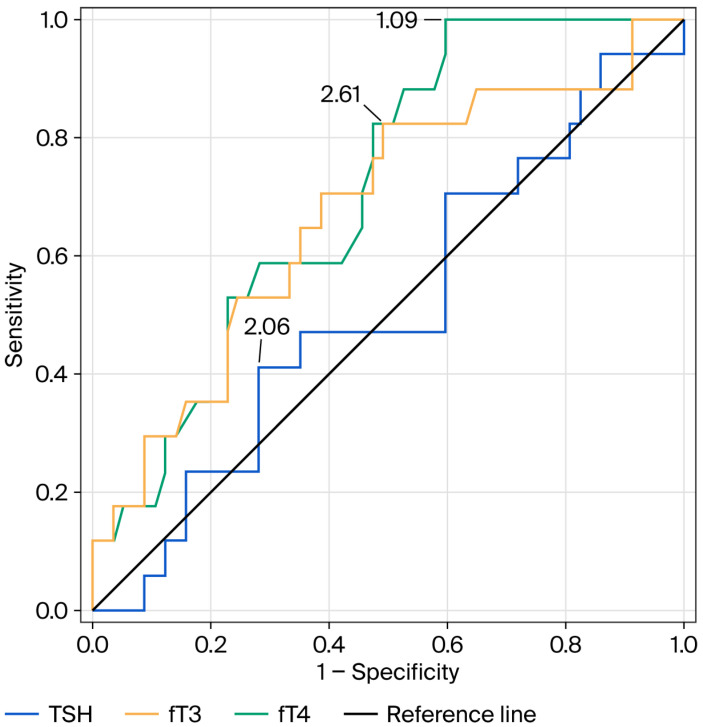
ROC analysis of FT3, FT4, and TSH serum concentrations in predicting death occurrence during follow-up in dialysis patients.

ROC curve analysis was conducted to assess the prognostic value of TH parameters (Table 5 and Figure 4). FT4 demonstrated the highest discriminatory capacity (AUC = 0.71, *p* = 0.0009), followed by FT3 (AUC = 0.67, *p* = 0.03), while TSH levels did not exhibit significant prognostic value (AUC = 0.51, *p* = 0.9).

## 4. Discussion

### 4.1. Summary of Key Findings

This prospective bi-center observational study demonstrated a high prevalence of NTIS among patients undergoing maintenance hemodialysis and confirmed its relevancy with adverse clinical outcomes. The most important findings of this study are as follows: NTIS was accompanied by significantly lower dialysis adequacy, as assessed by spKt/V. The introduction of stage-based NTIS classification revealed a graded dose–response relationship between NTIS severity and declining dialysis adequacy. Surprisingly, NTIS severity was associated with differences in pulmonary comorbidities; however, this requires confirmation in further research. Additionally, reduced serum fT3 and fT4 concentrations were associated with increased risk of death, and clinically applicable cut-off values for these hormones were identified.

### 4.2. NTIS and Dialysis Adequacy—Clinical and Methodological Implications

Alterations in TH metabolism are common in patients with advanced CKD and ESRD [3,8,17,20,26]. Reduced serum triiodothyronine concentrations with normal or low-normal TSH levels represent the most characteristic biochemical patterns and are considered hallmarks of NTIS in this population [2,3,24]. The high prevalence of NTIS observed in our cohort is consistent with previous reports and supports the concept that NTIS reflects systemic metabolic and inflammatory stress rather than primary thyroid disease [6,24].

A central observation of this study was the association between NTIS and reduced dialysis adequacy. Dialysis adequacy, measured using the Kt/V ratio, remains a cornerstone of hemodialysis quality assessment and a well-established determinant of survival [27,39,40].

In the present study, patients with NTIS had lower spKt/V values than euthyroid patients. To our knowledge, no previous study has directly examined the association between the NTIS and dialysis adequacy, underscoring the originality of this finding.

Although the difference in median spKt/V between groups reached statistical significance, the absolute difference was relatively small (1.1 vs. 1.0). Therefore, the clinical significance of this finding should be interpreted with caution. It is possible that the observed association reflects differences in overall clinical or metabolic status. However, note that, according to the literature, even a small increase of 0.1 in the Kt/V ratio results in a decrease in mortality among patients by approximately 7% [41]. The low statistical significance in our study could have been due to the small size of our group.

No statistical significance was demonstrated in terms of dialysis duration between groups. However, among patients with NTIS, the median dialysis duration was longer than that in the euthyroid group, which may be considered a confounding factor in the obtained results. The analysis of the NTIS group vs. the euthyroid group and survivors vs. non-survivors did not reveal a statistically significant difference in hemoglobin concentration. This finding is not consistent with the results of studies in the literature reporting that anemia is associated with worse clinical outcomes in HD patients [42]. The discrepancy between our results and those in the literature may be due to our cohort’s small size. Patients in the NTIS group were taking higher doses of ESA, which, as is known, is associated with increased mortality in ESRD patients [43].

It should be emphasized that additional research accounting for more possible confounding factors are necessary.

### 4.3. NTIS Severity and Dose–Response Relationship with Kt/V

By applying stage-based NTIS classification, we demonstrated a graded decline in spKt/V with increasing NTIS severity. Patients with NTIS stage III disease exhibited significantly lower dialysis adequacy than those with stage I disease, suggesting a dose–effect relationship. This observation extends previous research that assessed NTIS primarily as a binary condition and failed to identify associations with dialysis adequacy [29,44]. Our findings indicate that NTIS severity may reflect the magnitude of the metabolic burden and disease complexity in ESRD. A significant limitation of these results is the lack of consideration of biochemical data related to nutritional status, as well as the lack of inflammatory parameters in patients with ESRD. NTIS is known to correlate with malnutrition and inflammation, and according to the literature, the decrease in TH in dialysis patients may be a marker of both malnutrition and inflammation, which should be considered as a confounding factor in our study [29].

Another observation is the relationship between NTIS severity and pulmonary comorbidities. Patients with advanced NTIS were characterized by a lower prevalence of pulmonary diseases than those in earlier stages, a finding that is not readily explained by existing data. However, subgroup analyses examining associations between NTIS severity and pulmonary comorbidities were based on small subgroup sizes and sparse contingency tables. For this reason, these findings should be considered hypothesis-generating and interpreted with caution. Although NTIS is widely recognized as a systemic adaptive response to chronic illness, inflammation, and metabolic stress [1,2,6,17], its association with pulmonary comorbidities has not been systematically investigated in patients undergoing hemodialysis.

In ESRD patients, both NTIS and pulmonary dysfunction may reflect overlapping pathophysiological mechanisms, including chronic inflammation, protein–energy wasting, impaired muscle function, and a catabolic state [28,29,30,45,46]. Thyroid hormones, particularly triiodothyronine, influence respiratory muscle performance and tissue metabolism. Low T3 levels are considered markers of systemic illness severity rather than organ-specific pathology [6,24]. The authors emphasize that this result requires confirmation in larger independent cohorts.

### 4.4. Mineral and Bone Metabolism Markers and NTIS

Disturbances in mineral and bone metabolism were highly prevalent in the study population, reflecting advanced ESRD [26,47,48]. However, no significant differences were observed between the euthyroid and NTIS groups regarding calcium, phosphate, or PTH concentrations. This suggests that NTIS represents a metabolic phenotype that is largely independent of bone–mineral disorders. The higher urea concentrations and lower Kt/V values observed in advanced NTIS stages indicate that metabolic disturbances may be secondary to dialysis inadequacy and catabolic burden, rather than the primary dysregulation of mineral metabolism.

### 4.5. Thyroid Hormones and Risk of Deaths

An association between low TH levels and increased mortality in patients undergoing dialysis has been consistently reported [23,35,36,48,49,50]. In line with these findings, non-survivors in our cohort exhibited significantly lower serum fT3 and fT4 concentrations, whereas TSH did not demonstrate prognostic value. These results support the concept that peripheral TH levels provide more relevant prognostic information than pituitary-derived parameters in patients with ESRD.

We also aimed to identify clinically applicable cut-off values for fT3 and fT4 levels in this study. The fT3 cut-off identified in our study was comparable to the values proposed in previous research [51], whereas fT4 demonstrated high sensitivity for risk of death prediction. Differences between studies may be related to heterogeneous populations, laboratory methodologies, and the influence of nutritional and inflammatory status [52].

### 4.6. Clinical Implications and Conceptual Framework

Collectively, our findings support the concept of a “vicious circle” linking NTIS, reduced dialysis adequacy, and adverse outcomes. This observational study cannot determine whether inadequate dialysis contributes to NTIS through persistent uremic and inflammatory stress or whether NTIS reflects a systemic state that impairs dialysis effectiveness and organ function. Nevertheless, routine assessment of TH levels may aid in identifying patients at risk of inadequate dialysis and increased risk of death and may complement traditional technical measures of dialysis quality.

Although causality cannot be inferred from this observational study, our findings raise the hypothesis that NTIS may identify patients in whom standard dialysis dosing may be insufficient. Thus, further prospective interventional studies are warranted to determine if optimizing dialysis prescription, including treatment time or dialysis modality, could influence thyroid hormone profiles and clinical outcomes.

Further studies could investigate the association between mean thyroid hormone measurements (over a longer follow-up period) and the adequacy of dialysis therapy while accounting for possible confounding factors such as micro-inflammatory status, protein malnutrition, selenium deficiency, and deiodinase gene polymorphisms in a large cohort of ESRD patients.

### 4.7. Limitations

This study has some limitations: The sample size was modest. A limitation of our study is the lack of information regarding potential confounding factors, such as microinflammation, protein malnutrition, nutritional status, or selenium deficiency among ESRD patients. Hemodialysis patients have a large number of comorbidities that contribute to NTIS, so the vicious circle of disorders makes it difficult to clearly establish a clear cause-and-effect relationship.

Another limitation was the short period of observation. Thyroid function was assessed at a single time point, and multivariate analyses were not feasible. NTIS is a dynamic phenomenon in dialysis patients. Therefore, further research with averaged measurement values of TH and a longer observation time is necessary, as they could increase the accuracy of the results.

The pulmonary comorbidities were assessed clinically rather than using functional testing. Dialysis adequacy was assessed using spKt/V, which does not account for post-dialysis urea rebound. Furthermore, due to the inconvenience of 24 h urine collection for our patients, we did not include data on residual diuresis, which may determine solute clearance and endocrine disruptions.

## 5. Conclusions

Non-thyroidal illness syndrome is prevalent among patients undergoing hemodialysis and appears to be associated with reduced dialysis adequacy and increased risk of deaths. NTIS severity correlates with a progressive decline in spKt/V, indicating a gradational phenomenon independent of mineral–bone metabolism disorders. Reduced fT3 and fT4 concentrations identify patients at a higher risk of adverse outcomes and may serve as clinically useful markers for risk stratification in the hemodialysis population. Thus, the presence and severity of NTIS may therefore serve as a clinical signal, prompting closer evaluation of dialysis adequacy and overall treatment effectiveness in hemodialysis patients. However, the results should be confirmed on a larger sample.

## Figures and Tables

**Table 1:** Biochemical blood parameters: methodology and range of reference values.

Laboratory Parameters	Reference Range	Assessment Method
TSH [uIU/mL]	0.55–4.78	chemiluminescent immunoassay CLIA
FT4 [ng/dL]	0.89–1.76	
FT3 [pg/mL]	2.30–4.20	
Ca [mmol/L]	2.25–2.75	spectrophotometry
P [mmol/L]	0.8–1.6	
PTH [pg/mL]	10–60	chemiluminescent immunoassay CLIA
Anti-TPO [IU/mL]	<40	
Anti-TG [IU/mL]	<110	
sCr [µmol/L]	M: 53–97	
	W: 44–71	
Urea [mmol/L]	2–6.7	
Hemoglobin [g/dL]	M: 13–17.5	absorption photometry
	W: 12–16	

Abbreviations: TSH, thyrotropin; FT4, thyroxine; FT3, triiodothyronine; Ca, calcium, P, phosphate, PTH, parathyroid hormone; anti-TPO, anti-thyroid peroxidase antibodies; anti-TG, anti-thyroglobulin antibodies; sCr, serum creatinine; CLIA, clinical laboratory.

**Table 2:** Clinical and laboratory profiles of participants.

Patients with ESRD, *n* = 74			
Variable	Euthyroid *n* = 37	NTIS *n* = 37	*p*-Value
	Demographics, *n* (%) or median (min–max)		
Age, years	68 (18–89)	65 (18–88)	0.5 *
Body weight, kg	77 (38–116)	75 (29–108)	0.8 *
BMI	27 (17–35)	26.5 (13–37)	0.8 *
Sex, female	14 (37.8%)	15 (40.5%)	0.8 ^#^
Heart disease			
Yes	31 (83.8%)	33 (89.2%)	0.7 ^^^
MD	2 (5.4%)	0	
T2DM,			
Yes	7 (18.9%)	11 (29.7%)	0.3 ^#^
MD	1 (2.7%)	0	
Pulmonary comorbidities			
Yes	4 (10.8%)	5 (13.5%)	0.9 ^^^
MD	1 (2.7%)	0	
Dialysis duration, months	28 (3–208)	39 (3–288)	0.3 *
Use of ESA (%)	32 (86.5%)	31 (83.8%)	0.7 ^#^
ESA dose (IU/week) a	7500 (3000–21,000)	9000 (3000–18,000)	0.9 *
ESA dose (IU/week) b	9000 (3000–18,000)	10,500 (3000–12,000)	0.9 *
ESA dose (ug/week) c	30 (10–60)	30 (10–50)	0.8 *
	Laboratory parameters, median (min–max)		
Ca	2.2 (1.6–2.6)	2.1 (1.7–3.1)	0.3 *
P	1.7 (0.8–3.7)	2.0 (0.8–5.6)	0.4 *
PTH	314.5 (23.7–1606)	359 (35–1773)	0.6 *
Anti-TPO	35 (9–102)	31.5 (10–1271)	0.7 *
Anti-TG	24 (1.3–348)	101 (1.3–1000)	0.6 *
spKt/V ratio	1.1 (0.7–1.8)	1.0 (0.6–1.4)	0.03 *
sCr, [µmol/L] before HD	765.5 (180.3–1219.9)	808.9 (283.8–1502.8)	0.3 *
Urea [mmol/L] before HD	19.8 (4.7–34.3)	22 (10.2–41.5)	0.2 *
sCr, [µmol/L] after HD	308.5 (84.9–598.5)	351.8 (146.7–1064.3)	0.1 *
Urea [mmol/L] after HD	7.2 (3.2–15.0)	8.0 (4.1–21.5)	0.1 *
Hemoglobin [g/dL]	10.7 (8.5–14.1)	10.5 (8.0–14.2)	0.2 *

Abbreviations: ESRD, end-stage renal disease; BMI, body mass index; T2DM, diabetes mellitus type 2; ESA, erythropoiesis-stimulating agent; ESA a, epoetin alfa; ESA b, epoetin beta; ESA c, darbepoetin alfa; Ca, calcium; P, phosphate; PTH, parathyroid hormone; anti-TPO, anti-thyroid peroxidase antibodies; anti-TG, anti-thyroglobulin antibodies; spKt/V, ratio used to measure hemodialysis adequacy; HD, hemodialysis; sCr, serum creatinine; median (min–max) min, minimum; max, maximum; *, Mann–Whitney U test; ^#^, Pearson’s chi-square test; ^^^, chi-square test with Yates’s continuity correction. Statistical significance was set at *p* < 0.05; MD, missing data.

**Table 3:** Comparison of the demographic, clinical, and laboratory characteristics of ESRD patients according to NTIS stage (stage I vs. stage III). Data are presented as *n* (%) or the median (min–max).

	Patients with ESRD		
Variables	NTIS Stage I *n* = 14	NTIS Stage III *n* = 23	*p*-Value
	Demographics, *n* (%) or median (min–max)		
Age, years	71 (60–85)	58 (18–88)	0.01 *
Body weight (kg)	74.5 (63–108)	75 (29–106)	0.9 *
BMI	27 (21–37)	26.5 (13–32)	0.3 *
Sex, female	7 (50%)	8 (34.8%)	0.4 ^#^
Heart disease, yes	14 (100%)	19 (82.6%)	0.3 ^^^
T2DM, yes	3 (21.4%)	8 (34.8%)	0.6 ^^^
Pulmonary comorbidities, yes	5 (35.7%)	0	0.01 ^^^
Dialysis duration, months	29.5 (3–288)	48 (7–146)	0.7 *
Use of ESA (%)	13 (41.9%)	18 (58.1%)	0.5 ^^^
ESA dose (IU/week) a	3000 (3000–3000)	12,000 (3000–18,000)	-
ESA dose (IU/week) b	10,500 (3000–12,000)	-	-
ESA dose (ug/week) c	20 (10–40)	30 (20–50)	0.4 *
	Laboratory parameters, median (min–max)		
Ca	2.1 (1.8–2.5)	2.1 (1.7–3.1)	0.4 *
P	1.8 (0.8–5.7)	2.2 (0.9–3.1)	0.4 *
PTH	257.5 (35–929)	402.8 (113–1773)	0.1 *
spKt/V ratio	1.1 (0.6–1.4)	0.9 (0.7–1.3)	0.008 *
sCr, [µmol/L] before HD	821.7 (388.9–1061.7)	792.9 (283.8–1502.8)	0.6 *
Urea [mmol/L] before HD	17.9 (10.2–30.5)	24.2 (13.0–41.5)	0.03 *
sCr, [µmol/L] after HD	302.8 (146.7–452.6)	368.6 (146.7–1064.3)	0.2 *
Urea [mmol/L] after HD	6.8 (4.1–11.0)	9.5 (4.2–21.5)	0.02 *
Hemoglobin [g/dL]	10.1 (8.8–12.1)	10.6 (8–14.2)	0.5 *

Abbreviations: ESRD, end-stage renal disease; BMI, body mass index; T2DM, diabetes mellitus type 2; ESA, erythropoiesis-stimulating agent; ESA a, epoetin alfa; ESA b, epoetin beta; ESA c, darbepoetin alfa, Ca, calcium; P, phosphate; PTH, parathyroid hormone; sCr, serum creatinine; spKt/V, ratio used to measure the adequacy of hemodialysis; HD, hemodialysis; median (min–max), where min indicates minimum and max indicates maximum. * Mann–Whitney U test. ^#^ Pearson’s chi-square test. ^^^ Chi-square test with Yates’s continuity correction. Statistical significance was set at *p* < 0.05.

**Table 4:** Comparison of survivors and non-survivors among dialysis patients with respect to selected clinical and laboratory parameters, including serum hormone concentrations.

Patients with ESRD			
Variable	Survivors *n* = 57	Non-Survivors *n* = 17	*p*-Value
	Demographics, *n* (%) or median (min–max)		
Age (years)	66 (18–89)	69 (25–89)	0.7 *
Body weight (kg)	78 (38–116)	74 (29–92)	0.9 *
Body mass index	27 (17–37)	24 (13–31)	0.3 *
Sex, female	21 (36.8%)	8 (47.1%)	0.4 ^#^
Heart disease, yes	49 (87.5%)	15 (93.7%)	0.8 ^^^
T2DM, yes	12 (21.1%)	6 (37.5%)	0.2 ^#^
Pulmonary comorbidities, yes	8 (14.1%)	1 (6.25%)	0.7 ^^^
Dialysis duration, months	28 (3–288)	57 (5–187)	0.2 *
Use of ESA (%)	51 (89.5%)	12 (70.6%)	0.06 ^#^
ESA dose (IU/week) a	6000 (3000–18,000)	15,000 (9000–21,000)	0.3 *
ESA dose (IU/week) b	9000 (3000–18,000)	6000 (3000–12,000)	0.4 *
ESA dose (ug/week) c	30 (10–60)	30 (10–40)	0.9 *
	Laboratory parameters, median (min–max)		
Ca	2.1 (1.6–2.6)	2.1 (1.7–3.1)	0.7 *
P	1.9 (0.8–5.7)	1.7 (0.8–2.9)	0.3 *
PTH	350 (35–1773)	234 (23.7–1087)	0.2 *
Kt/V ratio	1.06 (0.68–1.7)	1.08 (0.64–1.38)	0.1 *
sCr [µmol/L] before HD	819.5 (180.3–1502.8)	558.7 (186.5–1177.5)	0. 05 *
Urea [mmol/L] before HD	21.8 (4.7–41.5)	18.8 (14.9–28.2)	0.5 *
sCr [µmol/L] after HD	338.6 (84.8–1064.3)	269.6 (111.4–465.9)	0.6 *
Urea [mmol/L] after HD	7.3 (3.2–21.5)	8.0 (5.0–16.2)	0.7 *
TSH	1.5 (0.35–4.86)	1.4 (0.29–2.67)	0.9 *
fT3	2.62 (1.76–3.48)	2.30 (1.12–3.1)	0.03 *
fT4	1.01 (0.61–1.39)	0.85 (0.59–1.09)	0.008 *
Hemoglobin [g/dL]	10.6 (8.1–14.2)	10.5 (8.0–14.1)	0.5 *

Abbreviations: * Mann–Whitney U test; ^#^ Pearson’s chi-square test; ^^^ Chi-square test with Yates’s continuity correction. Statistical significance was set at *p* < 0.05.

**Table 5:** ROC analysis of TSH, fT3, and fT4 concentrations in predicting death occurrence during follow-up in dialysis patients.

Parameter	Cut-Off Value	Sensitivity	Specificity	AUC	*p*-Value
TSH (uIU/mL)	2.06	0.41	0.72	0.51	0.9
fT3 (pg/mL)	2.61	0.82	0.51	0.67	0.03
fT4 (ng/dL)	1.09	1	0.40	0.71	0.0009

## Data Availability

The data presented in this study are available upon request from the corresponding author but are not publicly available due to privacy.

## Abbreviations

The following abbreviations are used in this manuscript:

AUC	Area under the curve
NTIS	Non-thyroidal illness syndrome
fT3	Free triiodothyronine
fT4	Free thyroxine
BMI	Body mass index
DA	Dopamine
ESA	Erythropoiesis-stimulating agent
ESRD	End-stage renal disease
HD	Hemodialysis
PTH	Parathyroid hormone
ROC	Receiver operating characteristic
HPT	Hypothalamic–pituitary–thyroid
TSH	Thyroid-stimulating hormone
TH	Thyroid hormone

## Appendix A

### Appendix A.1. Supplementary Methods

#### Appendix A.1.1. Sample Size Considerations and Statistical Power

This study was designed as an exploratory prospective observational analysis and included all eligible maintenance hemodialysis patients treated in the two participating centers during the recruitment period (n = 74). Therefore, the final sample size was determined by the available patient population rather than by an a priori calculation for a single predefined primary endpoint.

The analyses addressed three related but methodologically distinct questions:

- differences in dialysis adequacy (spKt/V) between euthyroid and NTIS patients,
- the discriminatory ability of thyroid hormone parameters for mortality status during follow-up (ROC analysis),
- associations between NTIS severity and pulmonary comorbidities.

#### Appendix A.1.2. ROC Analysis for the Risk of Death

The cohort included 74 patients, of whom 17 died and 57 survived during the 6-month follow-up period. For the ROC analyses, the observed areas under the curve (AUC) were 0.71 for fT4 and 0.67 for fT3.

Using the asymptotic standard error approach of Hanley and McNeil, the approximate statistical power to detect AUC values greater than 0.50 at α = 0.05 was estimated as follows:

**Marker**	**AUC**	**Cases/Controls**	**Approx. z**	**Approx. Power**
fT4	0.71	17/57	2.73	~78%
fT3	0.67	17/57	2.15	~58%

These estimates indicate that the available number of events was sufficient for exploratory discrimination analysis, particularly for fT4. However, the number of deaths was not sufficient for formal validation of prognostic cut-off values or for stable multivariable prognostic modelling.

#### Appendix A.1.3. Differences in Dialysis Adequacy (spKt/V)

For the comparison of spKt/V between euthyroid and NTIS patients (37 vs. 37), the observed *p* value in the Mann–Whitney test was *p* = 0.03. This corresponds approximately to an effect size r ≈ 0.25, equivalent to a Cohen’s d of approximately 0.5.

For two groups of this size, the approximate statistical power at α = 0.05 is around 60%. This indicates a moderate signal that should be interpreted cautiously and confirmed in larger cohorts.

#### Appendix A.1.4. NTIS Severity and Pulmonary Comorbidities

Subgroup analyses examining associations between NTIS severity and pulmonary comorbidities were based on small subgroup sizes and sparse contingency tables. For this reason, these findings should be considered hypothesis-generating and interpreted with caution.

#### Appendix A.1.5. Summary

Overall, the available sample size and number of events were sufficient to support exploratory analyses and ROC discrimination assessment. However, the study was not designed to formally validate mortality cut-off values or to perform multivariable prognostic modelling. These results therefore require confirmation in larger independent cohorts.

## References

[1] Fliers E., Bianco A.C., Langouche L., Boelen A. (2015). Thyroid function in critically ill patients. Lancet Diabetes Endocrinol..

[2] Fliers E., Boelen A. (2021). An update on non-thyroidal illness syndrome. J. Endocrinol. Investig..

[3] Song S.H., Kwak I.S., Lee D.W., Kang Y.H., Seong E.Y., Park J.S. (2009). The prevalence of low triiodothyronine according to the stage of chronic kidney disease in subjects with a normal thyroid-stimulating hormone. Nephrol. Dial. Transplant..

[4] Sciacchitano S., Capalbo C., Napoli C., Anibaldi P., Salvati V., De Vitis C., Mancini R., Coluzzi F., Rocco M. (2022). Nonthyroidal Illness Syndrome: To Treat or Not to Treat? Have We Answered the Question? A Review of Metanalyses. Front. Endocrinol..

[5] Ortiga-Carvalho T.M., Chiamolera M.I., Pazos-Moura C.C., Wondisford F.E. (2016). Hypothalamus-Pituitary-Thyroid Axis. Compr. Physiol..

[6] de Vries E.M., Fliers E., Boelen A. (2015). The molecular basis of the non-thyroidal illness syndrome. J. Endocrinol..

[7] Boelen A., van der Spek A.H., Bloise F., de Vries E.M., Surovtseva O.V., van Beeren M., Ackermans M.T., Kwakkel J., Fliers E. (2017). Tissue thyroid hormone metabolism is differentially regulated during illness in mice. J. Endocrinol..

[8] Iglesias P., Díez J.J. (2009). Thyroid dysfunction and kidney disease. Eur. J. Endocrinol..

[9] Segarra A.B., Ramírez M., Banegas I., Hermoso F., Vargas F., Vives F., Alba F., de Gasparo M., Prieto I. (2006). Influence of thyroid disorders on kidney angiotensinase activity. Horm. Metab. Res..

[10] Del Compare J.A., Aguirre J.A., Ibarra F.R., Barontini M., Armando I. (2001). Effects of thyroid hormone on the renal dopaminergic system. Endocrine.

[11] Michael U.F., Barenberg R.L., Chavez R., Vaamonde C.A., Papper S. (1972). Renal handling of sodium and water in the hypothyroid rat. Clearance and micropuncture studies. J. Clin. Investig..

[12] Lin H.H., Tang M.J. (1997). Thyroid hormone upregulates Na, K-ATPase alpha and beta mRNA in primary cultures of proximal tubule cells. Life Sci..

[13] Lossow K., Renko K., Schwarz M., Schomburg L., Schwerdtle T., Kipp A.P. (2021). The Nutritional Supply of Iodine and Selenium Affects Thyroid Hormone Axis Related Endpoints in Mice. Nutrients.

[14] Peeters R.P., Kester M.H., Wouters P.J., Kaptein E., van Toor H., Visser T.J., Van den Berghe G. (2005). Increased thyroxine sulfate levels in critically ill patients are a result of decreased hepatic type I deiodinase activity. J. Clin. Endocrinol. Metab..

[15] Cotoi L., Borcan F., Sporea I., Amzar D., Schiller O., Schiller A., Dehelean C.A., Pop G.N., Borlea A., Stoian D. (2020). Thyroid Pathology in End-Stage Renal Disease Patients on Hemodialysis. Diagnostics.

[16] Lo J.C., Chertow G.M., Go A.S., Hsu C.Y. (2005). Increased prevalence of subclinical and clinical hypothyroidism in persons with chronic kidney disease. Kidney Int..

[17] Lim V.S. (2001). Thyroid function in patients with chronic renal failure. Am. J. Kidney Dis..

[18] Chonchol M., Lippi G., Salvagno G., Zoppini G., Muggeo M., Targher G. (2008). Prevalence of subclinical hypothyroidism in patients with chronic kidney disease. Clin. J. Am. Soc. Nephrol..

[19] Naseem F., Mannan A., Dhrolia M.F., Imtiaz S., Qureshi R., Ahmed A. (2018). Prevalence of subclinical hypothyroidism in patients with chronic kidney disease on maintenance hemodialysis. Saudi J. Kidney Dis. Transplant..

[20] Kim E.O., Lee I.S., Choi Y.A., Lee S.J., Chang Y.K., Yoon H.E., Jang Y.S., Lee J.M., Kim H.S., Yang C.W. (2013). Unresolved subclinical hypothyroidism is independently associated with progression of chronic kidney disease. Int. J. Med. Sci..

[21] Tsuda S., Nakayama M., Matsukuma Y., Yoshitomi R., Haruyama N., Fukui A., Nakano T., Tsuruya K., Kitazono T. (2021). Subclinical hypothyroidism is independently associated with poor renal outcomes in patients with chronic kidney disease. Endocrine.

[22] Brough R., Jones C. (2006). Iatrogenic iodine as a cause of hypothyroidism in infants with end-stage renal failure. Pediatr. Nephrol..

[23] Meuwese C.L., Dekker F.W., Lindholm B., Qureshi A.R., Heimburger O., Barany P., Stenvinkel P., Carrero J.J. (2012). Baseline Levels and Trimestral Variation of Triiodothyronine and Thyroxine and Their Association with Mortality in Maintenance Hemodialysis Patients. Clin. J. Am. Soc. Nephrol..

[24] Wartofsky L., Burman K.D. (1982). Alterations in thyroid function in patients with systemic illness: The ‘euthyroid sick syndrome’. Endocr. Rev..

[25] Chávez Valencia V., Mejía Rodríguez O., Viveros Sandoval M.E., Bermúdez J.A., Castellanos S.G., de la Cruz C.O., Córdova M.A.R. (2018). Prevalence of malnutrition-inflammation complex syndrome and its correlation with thyroid hormones in chronic haemodialysis patients. Nefrologia (Engl. Ed.).

[26] Rhee C.M., Nissenson A.R., Fine R., Mehrotra R., Zaritsky J. (2016). Abnormalities of Thyroid Function in Chronic Dialysis Patients. Handbook of Dialysis Therapy.

[27] Gotch F.A., Sargent J.A. (1985). A mechanistic analysis of the National Cooperative Dialysis Study (NCDS). Kidney Int..

[28] Zoccali C., Tripepi G., Cutrupi S., Pizzini P., Mallamaci F. (2005). Low triiodothyronine: A new facet of inflammation in end-stage renal disease. J. Am. Soc. Nephrol..

[29] Fernández-Reyes M.J., Sánchez R., Heras M., Tajada P., García L., Iglesias P., García Arévalo M.C., Molina A., Rodríguez A., Alvarez-Ude F. (2009). Can FT_3_ levels facilitate the detection of inflammation or catabolism and malnutrition in dialysis patients?. Nefrologia.

[30] Yavuz D., Sezer S., Yavuz R., Canoz M.B., Altinoglu A., Elsurer R., Arat Z., Ozdemir F.N. (2014). Free triiodothyronine in hemodialysis patients: Link with malnutrition and inflammation. Iran. J. Kidney Dis..

[31] Kaptein E.M., Feinstein E.I., Nicoloff J.T., Massry S.G. (1983). Serum reverse triiodothyronine and thyroxine kinetics in patients with chronic renal failure. J. Clin. Endocrinol. Metab..

[32] Narasaki Y., Sohn P., Rhee C.M. (2021). The Interplay Between Thyroid Dysfunction and Kidney Disease. Semin. Nephrol..

[33] Rhee C.M. (2019). Thyroid disease in end-stage renal disease. Curr. Opin. Nephrol. Hypertens..

[34] Bello G., Ceaichisciuc I., Silva S., Antonelli M. (2010). The role of thyroid dysfunction in the critically ill: A review of the literature. Minerva Anestesiol..

[35] Zoccali C., Mallamaci F., Tripepi G., Cutrupi S., Pizzini P. (2006). Low triiodothyronine and survival in end-stage renal disease. Kidney Int..

[36] Xu H., Brusselaers N., Lindholm B., Zoccali C., Carrero J.J. (2016). Thyroid Function Test Derangements and Mortality in Dialysis Patients: A Systematic Review and Meta-analysis. Am. J. Kidney Dis..

[37] Molfino A., Beck G.J., Li M., Lo J.C., Kaysen G.A., FHN Investigators (2019). Association between change in serum bicarbonate and change in thyroid hormone levels in patients receiving conventional or more frequent maintenance haemodialysis. Nephrology.

[38] Yan W., Wang L., Huang T., Xu G. (2017). Treatment for non-thyroidal illness syndrome in advanced chronic kidney disease: A single-blind controlled study. J. Nephrol..

[39] National Kidney Foundation (2015). KDOQI clinical practice guideline for hemodialysis adequacy: 2015 update. Am. J. Kidney Dis..

[40] Daugirdas J.T. (2015). Kt/V (and especially its modifications) remains a useful measure of hemodialysis dose. Kidney Int..

[41] Held P.J., Port F.K., Wolfe R.A., Stannard D.C., Carroll C.E., Daugirdas J.T., Bloembergen W.E., Greer J.W., Hakim R.M. (1996). The dose of hemodialysis and patient mortality. Kidney Int..

[42] Foley R.N., Parfrey P.S., Harnett J.D., Kent G.M., Murray D.C., Barre P.E. (1996). The impact of anemia on cardiomyopathy, morbidity, and and mortality in end-stage renal disease. Am. J. Kidney Dis..

[43] Regidor D.L., Kopple J.D., Kovesdy C.P., Kilpatrick R.D., McAllister C.J., Aronovitz J., Greenland S., Kalantar-Zadeh K. (2006). Associations between changes in hemoglobin and administered erythropoiesis-stimulating agent and survival in hemodialysis patients. J. Am. Soc. Nephrol..

[44] Inaba M., Mori K., Tsujimoto Y., Yamada S., Yamazaki Y., Emoto M., Shoji T. (2021). Association of Reduced Free T3 to Free T4 Ratio with Lower Serum Creatinine in Japanese Hemodialysis Patients. Nutrients.

[45] Yang L., Wang M., Mo L., Yang Y., Cui Y., Wu Y. (2024). The relationship between sarcopenia and related bioindicators and changes after intensive lifestyle intervention in elderly East-China populations. BMC Musculoskelet. Disord..

[46] Carrero J.J., Qureshi A.R., Axelsson J., Yilmaz M.I., Rehnmark S., Witt M.R., Bárány P., Heimbürger O., Suliman M.E., Alvestrand A. (2007). Clinical and biochemical implications of low thyroid hormone levels (total and free forms) in euthyroid patients with chronic kidney disease. J. Intern. Med..

[47] Block G.A., Klassen P.S., Lazarus J.M., Ofsthun N., Lowrie E.G., Chertow G.M. (2004). Mineral Metabolism, Mortality, and Morbidity in Maintenance Hemodialysis. J. Am. Soc. Nephrol..

[48] Rodzoń-Norwicz M., Norwicz S., Sowa-Kućma M., Gala-Błądzińska A. (2023). Secondary hyperparathyroidism in chronic kidney disease: Pathomechanism and current treatment possibilities. Endokrynol. Pol..

[49] Fernández-Reyes M.J., Diez J.J., Collado A., Iglesias P., Bajo M.A., Estrada P., Del Peso G., Heras M., Molina A., Selgas R. (2010). Are low concentrations of serum triiodothyronine a good marker for long-term mortality in hemodialysis patients?. Clin. Nephrol..

[50] Ozen K.P., Asci G., Gungor O., Carrero J.J., Kircelli F., Tatar E., Sevinc Ok E., Ozkahya M., Toz H., Cirit M. (2011). Nutritional State Alters the Association between Free Triiodothyronine Levels and Mortality in Hemodialysis Patients. Am. J. Nephrol..

[51] Leśniak K., Rymarz A., Sobol M., Niemczyk S. (2023). Low Free Triiodothyronine as a More Sensitive Predictor of Survival Than Total Testosterone among Dialysis Men. Nutrients.

[52] Carrero J.J., Stenvinkel P. (2009). Persistent Inflammation as a Catalyst for Other Risk Factors in Chronic Kidney Disease: A Hypothesis Proposal. Clin. J. Am. Soc. Nephrol..

